# Potassium channels as molecular targets of endocannabinoids

**DOI:** 10.1080/19336950.2021.1910461

**Published:** 2021-07-20

**Authors:** Yu-Fung Lin

**Affiliations:** a Department of Physiology and Membrane Biology, University of California Davis, Davis, CA, USA; b Department of Anesthesiology and Pain Medicine, University of California Davis, Davis, CA, USA

**Keywords:** Potassium channels, endocannabinoid system, anandamide (AEA), 2-arachidonoylglycerol (2-AG), non-CB1, non-CB2 receptor-mediated

## Abstract

Endocannabinoids are a group of endogenous mediators derived from membrane lipids, which are implicated in a wide variety of physiological functions such as blood pressure regulation, immunity, pain, memory, reward, perception, reproduction, and sleep. *N*-Arachidonoylethanolamine (anandamide; AEA) and 2-arachidonoylglycerol (2-AG) represent two major endocannabinoids in the human body and they exert many of their cellular and organ system effects by activating the G_i/o_ protein-coupled, cannabinoid type 1 (CB1) and type 2 (CB2) receptors. However, not all effects of cannabinoids are ascribable to their interaction with CB1 and CB2 receptors; indeed, macromolecules like other types of receptors, ion channels, transcription factors, enzymes, transporters, and cellular structure have been suggested to mediate the functional effects of cannabinoids. Among the proposed molecular targets of endocannabinoids, potassium channels constitute an intriguing group, because these channels not only are crucial in shaping action potentials and controlling the membrane potential and cell excitability, thereby regulating a wide array of physiological processes, but also serve as potential therapeutic targets for the treatment of cancer and metabolic, neurological and cardiovascular disorders. This review sought to survey evidence pertaining to the CB1 and CB2 receptor-independent actions of endocannabinoids on ion channels, with an emphasis on AEA and potassium channels. To better understand the functional roles as well as potential medicinal uses of cannabinoids in human health and disease, further mechanistic studies to delineate interactions between various types of cannabinoids and ion channels, including members in the potassium channel superfamily, are warranted.

## INTRODUCTION

Ion channels are macromolecular pores in cell membranes that play a principal role in regulating the electrical properties of membranes and cellular excitability, thence enabling neurotransmission, sensory transduction, cognitive function, heart rhythm, motor movement, hormone secretion, reproduction, stress adaptation, and cell protection, processes crucial to sustain life. Ion channel proteins are subject to posttranslational modulation mediated by enzymes (e.g., kinases and phosphatases) and cellular messengers (e.g., Ca^2+^, Mg^2+^, protons, cGMP/cAMP, ATP/ADP, G proteins, reactive oxygen species [ROS], reactive nitrogen species [RNS], and phosphatidylinositol 4,5-bisphosphate [PIP_2_]). Notably, recent evidence has revealed that a variety of ion channel types are functionally modulated by cannabinoids [1–4], a structurally heterogeneous group of lipid-soluble compounds made naturally in plants (“phytocannabinoids”) or in animal cells (“endocannabinoids”) which have emerged as prominent modulators of diverse physiological and pathological processes.

Endocannabinoids refer to a class of signaling lipids consisting of amides, esters, and ethers of long-chain polyunsaturated fatty acids that are synthesized naturally from lipid precursors in plasma membranes of animal organisms [5]. Structurally distinct from the cannabinoids produced in the cannabis plant, endocannabinoids mimic the activity of Δ^9^-tetrahydrocannabinol (THC), the major psychoactive ingredient of cannabis [6]. Endocannabinoids are part of the endocannabinoid system that consists of endocannabinoids, their biosynthesizing and bio-degradative enzymes, the cannabinoid type 1 (CB1) and type 2 (CB2) receptors, and the cannabinoid uptake transporter. The endocannabinoid system functions as a homeostatic regulator in essentially all organ systems for physiological processes including neurodevelopment, synaptic transmission, learning and memory, nociception, stress and emotions, immunomodulation, hormone secretion, food intake and energy balance, digestive tract motility and secretion, reproductive function, and bone mass, among others [7–9]. In addition to the physiological roles, elements of the endocannabinoid system may serve as potential therapeutic targets in the pathological conditions [10]. Indeed, pharmacological manipulation of the endocannabinoid system has been shown to induce antinociceptive, anticonvulsive, anxiolytic, and anti-inflammatory effects, mitigating the symptoms or progression associated with different diseases [11].

**Figure 1.**
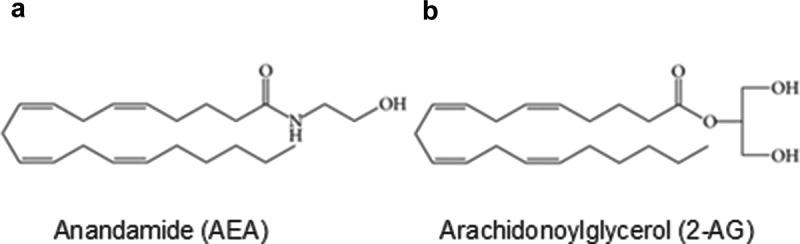
Chemical structures of *N*-arachidonoylethanolamine (AEA) and 2-arachidonoylglycerol (2-AG), two major endogenous cannabinoids (endocannabinoids). (a) AEA. (b) 2-AG

*N*-Arachidonoylethanolamine (anandamide; AEA) [12] (Figure 1a), 2-arachidonoylglycerol (2-AG) [13,14] (Figure 1b), and 2-arachidonylglyceryl ether (2-AGE; noladin ether) represent the most notable endocannabinoids [15]. AEA has widespread actions in the brain [15,16]: it influences learning and memory in the hippocampus, modulates locomotor activity, food intake, and the reward pathway in the basal ganglia and hypothalamus, and renders antinociception in the spinal cord and supra spinal sites. Besides the central nervous system, AEA is also produced in the peripheral tissues where it exerts local effects such as regulation of vascular tone and control of embryo transport/implantation in the female reproductive tract [for a review, see[8]]. While many of AEA’s effects involve activation of CB1 or CB2 receptors, pharmacological evidence nonetheless suggests that additional targets for cannabinoids exist. For example, orphan G protein-coupled receptors (e.g., GPR18, GPR55, GPR119), peroxisome proliferator-activated receptors (PPARs), equilibrative nucleoside transporter-1 (ENT-1; for adenosine reuptake in microglia), serotonin (5-HT) receptor subtypes, transient receptor potential (TRP) channels, and a variety of voltage-gated ion channels have all been suggested to mediate the effects of cannabinoids as their direct molecular targets [1,17–20]. The existence of additional targets of cannabinoids beyond CB1 and CB2 receptors reveals the complexity of the endocannabinoid system, manifesting an extensive endocannabinoid signaling network in homeostatic regulation and highlighting the promising prospect of targeting elements in this system for therapeutic interventions.

In this review, I summarize evidence concerning the canonical CB receptor-independent actions of endocannabinoids on ion channels, with a primary focus on the interactions between potassium channels and AEA, one of the principal endocannabinoids. The aim is to put into perspective open questions that remain to be addressed for better understanding of the physiological mechanisms as well as potential medicinal uses of cannabinoids in human health and disease.

## DISCUSSION

### Endocannabinoid interaction with potassium channels

The potassium channel superfamily exhibits a broad diversity that encompasses at least 70 channel members in mammals. Potassium channels are present in virtually all types of cells in all organisms, where they are involved in a multitude of physiological functions [for a review, see [21]]. These channels are important for setting the resting membrane potential, keeping fast action potentials short, terminating periods of intense activity, timing the interspike intervals during repetitive firing, and lowering the effectiveness of excitatory inputs on a cell [22]. Potassium channels are involved in regulation of neurotransmission, heart rate, muscle contraction, hormone release, and cell survival, among others, and hence represent attractive drug targets for the development of new therapeutic strategies for cancer as well as metabolic, neurological, and cardiovascular disorders [23–27].

The endocannabinoid AEA belongs to a class of signaling lipids consisting of amides of long-chain polyunsaturated fatty acids [15]. AEA is widely expressed in the human body and has been implicated in multiple physiological and pathophysiological processes, such as vascular tone, embryonic development, motor functions, pain reduction, cognition functions, sleep, immunomodulation, neuroprotection, cardioprotection, feeding and appetite, obesity, drug abuse, neurodegenerative diseases, and mood disorders [16, 28–30]. Many of AEA’s biological actions result from binding and activating the inhibitory G_i/o_ protein-coupled CB1 and CB2 receptors. For instance, CB1 receptor-mediated presynaptic depression likely involves activation of G protein-gated inwardly rectifying potassium (GIRK) and A-type (fast inactivating) potassium channels through a CB1 receptor-dependent signaling mechanism [for reviews, see [31,32]]. However, AEA may target ion channels and other proteins independently of CB1 or CB2 receptor activation to elicit its effects. Indeed, several members in the potassium channel superfamily may interact with cannabinoids to mediate non-CB1, non-CB2 receptor-dependent actions of these lipid messengers. In the following text, I will summarize findings supporting direct modulatory actions of the endocannabinoid AEA on potassium channels (Table 1) and on a few non-potassium channels as examples from other ion channel families (Table 2). The concentrations of cannabinoids shown in both Tables 1 and 2 represent EC_50_, IC_50_, or the range of concentrations being examined (numbers in parentheses) in individual studies.

**Table 1.** CB1/CB2 receptor-independent effects of endocannabinoids or analogs on potassium channels

**Cannabinoids**	**Channel targets**	**Changes in channel function**	**Cell models**		**IC_50_/EC_50_** **(Concentration range)^a^**	**Ref**
AEA/methAEA (indirect)	BK_Ca_	↑		HEK293 & Mouse aortic myocytes	(0.3–3.0 μM)	[36]
AEA	BK_Ca_	↑		EA.hy926 human endothelial-derived cell line	1.1 μM	[37]
NAGly	BK_Ca_	↑		Endothelial cells & Isolated mouse aorta	(0.1–30 μM)	[38]
AEA (extracellular)2-AGmethAEA	(h)CardiacKv4.3/KChIP2(≅ *I*_to, fast_)	↓↓↓		CHO & Human right atrial appendage myocytes	0.4 μM0.3 μM0.6 μM	[42]
AEA	*I*_to_	↓		Rat ventricular myocytes	(1–100 nM)	[44]
2-AG^b^	*I*_A_	↓		Mouse midbrain dopaminergic neurons	(0.03–30 μM)	[47]
AEA (either side ofmembrane)	Kv3.1 (non-inactivating)	↓ (into *I*_A_-type)		*Xenopus* oocytes &Rat hippocampal slices	(3 μM)(0.1–1 μM)	[61]
AEA (extracellular)	Kv1.2	↓		Murine fibroblasts(B82 cell line)	2.7 μM	[48]
AEA (extracellular)methAEA	Delayed rectifier Kv	↓		Rat aortic vascular smooth myocytes	0.6 μM (10 μM)	[50]
AEA (extracellular)2-AGmethAEA	Delayed rectifier Kv	↓		Rat primary cortical astrocytes & Neocortical slices	~0.3 μM (1 μM) (1 μM)	[51]
2-AG	Delayed rectifier Kv	↓		Mouse insulinoma R7T1 β-cells	20 μM	[54]
AEA (extracellular) & 2-AG	hKv1.5 *I*_SUS_ APD	↓ ↓↑		Mouse fibroblastsHuman atrial cellsMouse left atria	0.9–2.5 μM	[59]
AEA (cytoplasmic) (*as open channel blocker*)	hKv1.5	↓		HEK293	0.2 μM	[60]
2-AG (cytoplasmic)	K_ATP_	↓		Mouse insulinoma R7T1 β-cells	1 μM	[54]
AEA	K_ATP_ (cromakalin-induced)	↓		Follicle-enclosed *Xenopus* oocytes	8.1 μM	[29]
AEA^c^ (CB2 receptor-dependent)	K_ATP_	↑		Rat ventricular myocytes	(1–100 nM)	[44]
AEA/methAEAmethAEA	TASK-1 IKso	↓ ↓		COS-7, CHO & HEK293Cerebellar neurons	0.7 μM(10 μM)	[66]

^a^Numbers in parentheses denote the range of concentrations examined.

^b^This effect was measured at 37°C (in contrast to room temperature at which the recording experiment to determine the endocannabinoid effect was conducted in other studies listed in this table).

^c^This effect is CB2 receptor-dependent.

**Table 2.** CB1/CB2 receptor-independent effects of AEA on additional ion channel types

**Cannabinoids**	**Channel targets**	**Changes**	**Cell models**		**IC_50_/EC_50_(Concentration used)^a^**	**Ref**
***Ligand-gated ion channels***						
AEA	α1β1 GlyR	↑		*Xenopus* oocytes & VTA neurons	~250 nM	[86]
AEA	α2β3γ2 GABA_A_R	NS		*Xenopus* oocytes	(300 nM)	[86]
AEA	α7 nAChR	↓		*Xenopus* oocytes	229.7 nM	[85]
AEA	5-HT_3_R	↓		Rat nodose ganglion neurons	190 nM	[84]
***TRP channels***						
AEA	TRPV1	↑		HEK293 & oocytes (& rat perivascular sensory nerves)	5.3 μM	[83]
***Voltage-gated ion channels***						
AEA (direct, intracellular)	T-type Ca_V_	↓		HEK293; COS-7; CHO; NG 108–15	sub-μM	[87]
AEA	L-type Ca_V_	Displacing binding of Ca_V_ antagonists		Rabbit skeletal muscle membrane	4-30 μM	[88]
AEA	L-type Ca_V_ & Na_V_	↓		Rat ventricular myocytes	(1–10 μM)	[89]

This is not an exhaustive list.

^a^The number in parentheses denotes the concentration used.

#### Large-conductance, calcium-activated potassium channels

Large-conductance, calcium-activated potassium (BK) channels are present in most regions of the mammalian brain and in hormone-secreting cells where they modulate neurotransmitter and hormone release through co-localizing with voltage-gated calcium (Ca_V_) channels. In addition, BK channels are expressed in vascular smooth muscle cells where they contribute to the regulation of vascular contractile tone [21,33]. Moreover, BK channels may participate in the anticonvulsant and vasorelaxant effects of cannabinoids [34] and mediate cannabinoid-induced peripheral analgesia and firing-suppressing effects in primary sensory afferents after nerve injury [35]. Indeed, blocking BK channels reverses the firing-suppression effect of CB receptor agonists and the CB receptor agonist-induced peripheral analgesia [35].

With regard to the mechanism underlying (endo)cannabinoid-induced modulation of BK channels, it has been reported that the whole-cell current of BK channels acquired in both transfected human embryonic kidney 293 (HEK293) and native aortic cells is potentiated by the endocannabinoid AEA as well as methanandamide (methAEA), a synthetic, metabolically stable analog of AEA [36]. The potentiation of BK currents by AEA or methAEA is gradual, taking around 6–8 minutes to develop a peak response [36]. Notably, the BK-potentiating effect of methAEA is unaffected by a potent CB1 receptor antagonist AM251 or by pertussis toxin that prevents activation of G_i/o_ protein-coupled receptors, thus excluding an involvement of canonical CB1/CB2 receptors [36]. These results suggest that, by activating BK channels in vascular smooth muscle independently of CB1 and CB2 receptors, endocannabinoids may hyperpolarize membrane potential, reduce cell excitability, and consequently elicit vasodilation, providing neuroprotection after an ischemic stroke and/or suppressing excess activity of vascular smooth muscle tissues. However, the BK channel may not be a direct target of AEA/methAEA, as methAEA only enhances BK channel activity in whole-cell and cell-attached patch configurations but not in excised inside-out membrane patches [36]. On the other hand, Bondarenko et al. [37] have demonstrated that AEA concentration-dependently facilitates single BK channel activity in cell-free, inside-out patches obtained from human endothelial-derived EA.hy926 cells within a physiological Ca^2+^ range, which suggests that AEA directly interacts with and thereby modifies BK channel activity to induce endothelium-dependent vasorelaxation.

*N*‐arachidonoyl glycine (NAgly), an endogenous lipoamino acid structurally and metabolically related to the endocannabinoid AEA, also exhibits analgesic, anti‐inflammatory, and proinflammatory-resolving properties [5]. NAgly has been shown to activate BK channels in excised inside-out and outside-out patches obtained from human endothelial-derived EA.hy926 cells, and to cause BK channel blocker-sensitive membrane hyperpolarization in *in situ* mouse aortic endothelium, a critical event to initiate endothelium-dependent vasorelaxation [38]. This study identifies BK channels as cellular sensors for cannabinoids in *in vitro* and *in situ* endothelial cells, an effect that does not require activation of CB1/CB2 receptors or GPR18 (a postulated endothelial cannabinoid receptor), suggesting that NAgly initiates a CB1/CB2 receptor- and GPR18-independent activation of endothelial BK channels, which might contribute to vasodilation to cannabinoids [38]. Interestingly, the action of cannabinoids and cannabinoid-like compounds on endothelial cells is accompanied or at least partially underpinned by modulation of cholesterol level in caveolae, as the stimulatory response of endothelial BK channels to NAgly [38] or to AEA [37] is prevented following cholesterol depletion with methyl-β-cyclodextrin.

#### Voltage-gated potassium channels

Voltage-gated potassium (Kv) channels shape the action potential by controlling its repolarization phase and determine the membrane potential and duration of the interspike interval [21,22,39]. In general, delayed rectifier Kv channels function to keep single action potentials short and to permit high-frequency trains of action potentials, whereas rapidly inactivating A-type potassium channels space repetitive responses and help a cell fire at low frequencies [22].

1) A-type potassium channels:

A-type currents are calcium-independent Kv currents that undergo rapid activation and inactivation. A-type currents have been identified and characterized in neuronal, cardiac, vascular, genitourinary, and gastrointestinal smooth muscle cells. In the heart, potassium channels play important roles in determining the firing frequency in sinus node pacemaker cells as well as resting potential and the shape and duration of action potentials in cardiomyocytes [40]; A-type currents present in atrial and ventricular myocytes are referred to as “transient” outward current (*I*_to_). A complex formed by Kv4.2, Kv4.3, and KChIP2 may underlie the fast transient outward current (*I*_to, fast_) in cardiac muscle, while Kv1.4 may underlie a slower transient outward current (*I*_to, slow_) [21].

Endocannabinoids are involved in the regulation of cardiovascular function [41]. It has been demonstrated by Amorós et al. [42] that the whole-cell current of cloned human cardiac Kv4.3/KChIP2 channels (*I*_Kv4.3_; reproducing *I*_to, fast_ that plays a role in determining the height and the duration of phase 2 of the human cardiac action potential) expressed in stably transfected Chinese hamster ovary (CHO) cells is inhibited directly by AEA, 2-AG, and methAEA in a concentration-dependent fashion (IC_50_ ~ 0.3–0.6 μM). The inhibition is accompanied by accelerated inactivation and a hyperpolarization shift in the voltage dependence of inactivation [42]. Moreover, these inhibitory effects of endocannabinoids on *I*_Kv4.3_ are not mediated by activation of CB1/CB2 receptors or by modifications of the lipid order and microviscosity of the cell membrane; furthermore, the putative AEA-interacting site has been suggested to reside in the Kv4.3 α-subunit at its extracellular surface, as AEA and methAEA only block Kv4.3 channels when administered from the outside [42]. Human cardiac Kv4.3 channels thus represents a novel molecular target for AEA, 2-AG and cannabinoid analogues; the potent, direct inhibition of human cardiac *I*_Kv4.3_ and *I*_to, fast_ by AEA and 2-AG would increase the height and prolong the plateau duration of human cardiac action potential [42], altering human cardiac electrical activity.

Potassium channels are the basis for the change in action potential configuration in response to variation in heart rate and they are highly regulated [43]. Using isolated rat ventricular myocytes as a cell model, Li et al. [44] investigated the electrophysiological effects of AEA on K^+^ currents and reported that AEA concentration-dependently decreases the rapidly activating and inactivating transient outward current *I*_to_. Kinetically, AEA shifts the steady-state inactivation curve of *I*_to_ to the left and the recovery curve of *I*_to_ to the right, suggesting that AEA accelerates the voltage-dependent steady-state inactivation of *I*_to_ and suppresses *I*_to_ recovery from inactivation. The maximal effect of AEA on *I*_to_ develops slowly but can be measured within 8 min of initial exposure during continuous drug application [44]. Neither CB1 receptor antagonist AM251 nor CB2 receptor antagonist AM630 can abolish the inhibitory effect of AEA on *I*_to_, which further suggests that AEA reduces *I*_to_ through a non-CB1 and non-CB2 receptor-mediated mechanism [44]. In contrast, AEA exerts no effect on steady-state outward K^+^ current (proposed to reflect the activity of delayed rectifier potassium channels) and the inward rectifier current (*I*_K1_) in rat ventricular myocytes [44]. Cardiac *I*_to_ channels represent an important target of class III antiarrhythmic drugs, owing to their critical role in defining resting membrane potential, heart rate, and action potential shape and duration in cardiac sinus node cells and cardiac myocytes. Inhibition of *I*_to_ channels by AEA may therefore account for, at least in part, the antiarrhythmic action of AEA.

Endocannabinoids are also involved in the regulation of neuronal excitability [45]. In substantia nigra pars compacta (SNc) dopaminergic neurons, the fast inactivating A-type K^+^ current (*I*_A_) is mediated by Kv4.3 channels co-assembled with the accessory subunits KChIP3.1 [46]. Gantz and Bean [47] reported that at 37°C, *I*_A_ in isolated mouse midbrain dopaminergic neurons is inhibited by 2-AG (applied for 1 min or less in duration), the dominant endocannabinoid in the brain, in a concentration-dependent manner; moreover, this acute inhibitory effect of 2-AG on *I*_A_ does not require CB receptor activation or G protein signaling. The changes in A-type potassium channel gating caused by 2-AG include a shift of the voltage-dependent activation of *I*_A_ in the depolarizing direction, acceleration of the inactivation kinetics, and reduction of the maximal current for large depolarizations. The *I*_A_–inhibiting effect of 2-AG likely results from a direct action on the channel through a membrane lipid interaction; specifically, binding of 2-AG may modify the interaction of the voltage-sensing S4-S5 region of the channel with the S6 region that controls channel opening and closing [47]. It is thus suggested that by modulating *I*_A_, 2-AG and related lipid signaling molecules can directly tune neuronal excitability in a cell-autonomous manner [47].

2) Delayed rectifiers:

Besides exerting inhibitory effects on fast–inactivating potassium channels as described above, endocannabinoids also modulate the activity of classical delayed rectifier-type potassium channels. Classical delayed rectifiers like Kv1.2 (in the absence of β subunits), Kv2.1, and Kv3.1 do not show inactivation in the millisecond time scale; functionally, delayed rectifiers terminate action potentials, restore the dominant potassium permeability of the resting membrane potential, and shape the action potential [21]. AEA has been shown to inhibit *Shaker*-related Kv1.2 channels [48]; specifically, via accelerating the inactivation rate of Kv1.2, externally applied AEA concentration-dependently reduces Kv1.2 channel currents recorded at whole-cell and outside-out patch modes in fibroblasts stably transfected with Kv1.2 cDNA, whereas intracellularly dialyzed AEA is without effect. This inhibitory effect of AEA on Kv1.2 channels (IC_50_ = 2.7 μM) does not require activation of CB1 receptors or G protein signaling since neither CB1 receptor antagonists nor pertussis toxin could prevent the effect. Moreover, externally applied Δ^9^-THC, the major psychotropic constituent of cannabis, is capable of mimicking the inhibitory action of AEA on the Kv1.2 channel. Poling et al. [48] thus propose that an acceptor site on the extracellular side of the Kv1.2 channel recognizes AEA and other cannabinoid-like molecules.

In vascular smooth muscle cells, modulation of potassium channel activity plays an essential role in regulating membrane potential, which in turn influences the open probability of vascular Ca_V_ channels, the contractile tone of vascular smooth muscle, and blood flow [49]. External application of AEA has been reported to concentration-dependently (IC_50_ = 0.6 µM) reduce the delayed rectifier K^+^ current acquired in the whole-cell mode from freshly dissociated rat aortic smooth muscle cells in a CB1 receptor-independent manner [50]. In addition, methAEA (a metabolically stable analogue of AEA) and WIN 55,212–2 (a high-affinity CB1/CB2 receptor agonist) elicit similar inhibition of delayed rectifier K^+^ current, and these effects are also CB receptor-independent. Van den Bossche and Vanheel [50] thus concluded that cannabinoids likely bind to an external site on or near the delayed rectifier Kv channel of the aortic vascular smooth muscle cells to modulate the channel activity. Intriguingly, it appears that AEA may inhibit delayed rectifiers in different tissues via a similar mechanism, i.e., acting from the extracellular side of the membrane (see below).

It has been demonstrated that in both primary cultured rat cortical astrocytes and astroglial cells in cortical slices, low micromolar concentrations of AEA (and 2-AG) promote a strong reduction of the delayed rectifier outward K^+^ current (IC_50_ ~ 0.3 µM) [51]. Pharmacological blockade experiments further uncovered the AEA-induced inhibition is independent of CB1 receptor activation, AEA metabolism, and Ca^2+^ signaling. The delayed rectifier-inhibiting effect of AEA in astrocytes is mediated by its interaction with the extracellular leaflet of the plasma membrane based on the observation that only extracellularly, but not intracellularly, applied bovine serum albumin (BSA, a lipid scavenger) recovers the delayed rectifier K^+^ current suppressed by AEA. Moreover, the inhibitory effect of AEA on astrocyte delayed rectifier potassium channels does not involve an interaction of AEA with lipid rafts and/or caveolae as cholesterol-extracting agents fail to abrogate the AEA effect. Collectively, the findings made by Vignali et al. [51] suggest that AEA may stabilize the closed state of the delayed rectifier potassium channel by binding to hydrophobic determinants of the protein complex. Their findings support that endocannabinoids may effectuate their modulation of CNS function through regulating potassium channel-mediated homeostatic function of the astroglial syncytium, which might explain some non-neuronal effects of the endocannabinoid system (such as regulation of astroglial proliferation).

In the pancreas, Kv channels contribute to the regulation of insulin secretion by controlling the repolarization of pancreatic β-cell action potential [52]. The delayed rectifier is thought to be the dominant Kv current of β-cells [53], and hence has received much attention as potential therapeutic targets for type 2 diabetes [52]. It is noteworthy that delayed rectifier Kv channels in β-cells are also modulated by endocannabinoids. Spivak et al. [54] reported that 2-AG concentration-dependently inhibits the whole-cell current of delayed rectifier potassium channels in the mouse insulinoma cell line R7T1, with an IC_50_ of 20 μM; moreover, the inhibitory effect of 2-AG on β–cell delayed rectifier K^+^ current is CB receptor-independent [54]. The predominant delayed rectifier potassium channel in murine β-cells is the Kv2.1 type [52]; moreover, delayed rectifiers from Kv1 (such as Kv1.5), Kv2, and Kv3 subfamilies are also present in murine β-cells [52]. In addition, Kv1.5 is detected in human insulinoma cells [55] and is highly expressed in human islets [56]. It is possible that multiple distinct Kv channels comprise the delayed rectifier current of the human ß-cell [57].

In the human heart, Kv1.5 channels underlie the ultra-rapidly activating delayed rectifier K^+^ current (*I*_Kur_) that is critical for determining the height and duration of the human atrial action potential [58] and a potential target for treating atrial arrhythmias [21]. It has been demonstrated by Barana et al. [59] that both AEA and 2-AG exhibit a high potency (IC_50_ ≈ 0.9–2.5 μM) to inhibit human Kv1.5 (hKv1.5) current acquired in stably transfected mouse fibroblasts, an inhibitory effect that is independent of CB receptor activation and changes in the order and microviscosity of the membrane; in other words, the potencies of blockade are not related to the liposolubility of the compounds. Endocannabinoid-induced block of human cardiac Kv1.5 channels appears exclusively when endocannabinoids are applied at the external surface of the cell membrane; furthermore, the blockade by AEA or 2-AG is reduced by mutation of R487 located in the external vestibule-entryway, a residue that determines Kv1.5 sensitivity to external tetraethylammonium (TEA). AEA also inhibits atrial end pulse sustained K^+^ current (*I*sus) in human atrial myocytes which is mainly carried by Kv1.5 channels, and it significantly prolongs the duration of action potentials (APD) recorded in mouse left atria [59]. These findings thus support that endocannabinoids block human cardiac Kv1.5 channels via specific interaction at the extracellular TEA binding site (i.e., R487 located in the external entryway of the pore) of the channel, a mechanism by which the endocannabinoids regulate the shape of atrial action potentials [59].

Moreno-Galindo et al. [60] examined AEA modulation of cloned Kv1.5 channels expressed in transfected HEK293 cells and showed that AEA exerts high-potency block (IC_50_ ≈ 0.2 μM) of Kv1.5 channel currents in a CB receptor-independent manner from the cytoplasmic membrane surface, consistent with open-channel block. The postulated binding site for AEA in the study by Moreno-Galindo et al. [60] is located on the S6 domain that lines the channel vestibule, which, however, is distinct from the extracellularly located interaction site (i.e., R487 located in the external vestibule-entryway) suggested by Barana et al. [59]. Specifically, Moreno-Galindo et al. [60] identified Val505 and Ile508 within the S6 domain of Kv1.5 (both highly conserved among Kv family members), two residues facing toward the central cavity and constituting a motif that forms a hydrophobic ring around the ion conduction pathway, as AEA-interacting sites. Moreno-Galindo et al. [60] thus suggest that the hydrophobic ring motif may be a critical determinant of CB receptor-independent AEA modulation in other K^+^ channel families.

Studies reviewed above support that the endocannabinoid AEA may directly interact with delayed rectifier Kv channels and thereby modulate the channel function in a CB1/CB2 receptor-independent manner [48,50,51,59]. Interestingly, observations made on cloned Kv channels expressed in *Xenopus* oocytes have revealed that membrane lipids such as PIP_2_, by removing fast inactivation, can convert A-type potassium channels into delayed rectifiers, whereas AEA and arachidonic acid, by introducing fast inactivation, can convert non-inactivating delayed rectifiers into rapidly inactivating A-type potassium channels [61]. These findings thus imply that AEA may control the coding properties of neurons and synapses beyond the characteristics set by the expression profile of Kv channel protein subunits.

3) Rapidly activating delayed rectifiers:

The *ether-à-go-go*-related gene (ERG) potassium (i.e., Kv11) channel belongs to a voltage-activated potassium channel family encoded by three distinct gene subfamilies [62]. The human ERG (hERG) encodes the pore-forming subunit of the rapidly activating delayed rectifier potassium channel to give rise *I*_Kr_ that is important for repolarization of cardiac action potential [63]. The hERG potassium channel is also expressed in other tissues and cell types, including neural, smooth muscle and tumor cells [63]. Although how endocannabinoids modulate cardiac hERG channels has yet to be reported, AEA administrated at a concentration of 20 μM has been shown to mildly reduce the native ERG potassium current (*I*_K(erg)_) in clonal NG108-15 (mouse neuroblastoma x rat glioma hybrid) cells [64]. However, it remains to be determined whether the inhibitory effect of AEA on *I*_K(erg)_ requires activation of canonical CB receptors.

#### Two-pore domain potassium channels

In addition to BK and Kv channels, endocannabinoids also modulate two-pore domain potassium (K_2P_) channels. TWIK-related acid-sensitive potassium channel 1 (TASK-1), a member in the K_2P_ channel subfamily, encodes an acid- and anesthetic-sensitive background K^+^ current [21], which sets the resting membrane potential of both cerebellar granule neurons and somatic motoneurons, and may contribute to anesthetic-induced immobilization [65]. It has been demonstrated by Maingret et al. [66] that, unlike other K_2P_ channels, TASK-1 expressed in COS-7, CHO or HEK293 cells is directly blocked by submicromolar concentrations of AEA, an effect independent of both CB1 and CB2 receptors and G proteins. The inhibition of TASK-1 by AEA is specific, not mimicked by 2-AG, another endocannabinoid, or by Δ^9^-THC, the major psychoactive compound in cannabis; additionally, AEA hydrolysis is not involved, as the non-hydrolysable analogue methAEA is similarly effective [66]. AEA also blocks the standing-outward K^+^ current IKso (proposed to be principally carried by TASK-1 in cerebellar granule neurons [67]) and induces depolarization in cerebellar granule neurons [66]. These findings suggest that TASK-1 constitutes a novel, sensitive molecular target of AEA. It is recognized that cannabinoids including AEA profoundly affect locomotion, exerting a dose-related biphasic effect (i.e., stimulatory at low doses whereas depressant at high doses) [68,69]; direct modulation of TASK-1 by low doses of AEA might thus account for some of the biphasic, CB1 receptor-independent effects observed with AEA on locomotion.

#### Inwardly rectifying potassium channels

The role played by CB receptors in regulating energy balance is well established [70]. Endocannabinoid lipids are known to exert orexigenic effects via activation of central cannabinoid CB1 and CB2 receptors; in addition, the peripherally produced endocannabinoids also act as local regulators of insulin secretion through pancreatic β-cell CB receptor-mediated elevation in Ca^2+^ levels [71]. Beyond a physiological role in regulating energy balance, the cannabinoid transduction cascade may have a pathophysiological function to stimulate insulin-dependent lipid deposition through enhanced insulin output in response to increased levels of peripheral 2-AG, as seen in obesity [72], which implies that a dysregulated endocannabinoid system in the adipocytes and β-cells likely contributes to hyperlipidemia, hypoadiponectinemia, and hyperinsulinemia in obesity [72].

The ATP-sensitive potassium (K_ATP_) channel, a member in the inwardly rectifying potassium (Kir) channel subfamily [73], functions as a high-fidelity metabolic sensor that couples intracellular metabolic state to membrane excitability [74], serving a homeostatic role ranging from blood glucose regulation to cardioprotection [75–78]. K_ATP_ channels in pancreatic β-cells regulate insulin secretion in response to plasma glucose levels, via regulating membrane potential and thereby the activity of Ca_V_ channels, intracellular Ca^2+^ levels, and Ca^2+^-dependent exocytosis of insulin [79]. Interestingly, β-cell K_ATP_ channels have been shown to be modulated by endocannabinoids. Spivak et al. [54] reported that single-channel K_ATP_ currents acquired at 2 mM glucose in the inside-out patch configuration in R7T1 cells, a mouse insulinoma β-cell line, are concentration-dependently inhibited by 2-AG applied from the cytosolic side (IC_50_ = 1 μM). CB1 receptors are expressed in murine β-cells; however, the CB1 receptor antagonist AM251 does not affect 2-AG’s inhibitory action on K_ATP_ channel current, indicating that CB1 receptors do not mediate the effect [54]. The direct blockade of the K_ATP_ channel by 2-AG at low glucose concentrations would depolarize the β-cell and results in stimulation of insulin secretion; it is suggested that 2-AG may increase insulin secretion in a manner similar to that of the sulphonylureas (K_ATP_ channel blockers) by directly interacting with the K_ATP_ channel [54]. However, a role of AEA on the function of pancreatic β-cell K_ATP_ channels has yet to be explored.

Endogenous K_ATP_ channels in follicle-enclosed oocytes from *Xenopus laevis* are subject to modulation by gonadotropins [80] and may play important roles in oocyte maturation and hormonal regulation of oocyte development. The effect of endocannabinoids on cromakalim (a K_ATP_ channel opener)-activated K_ATP_ currents has been examined in follicular oocytes using two-electrode voltage-clamp recordings [29]. AEA inhibits cromakalim-activated K_ATP_ currents in a noncompetitive manner, with an IC_50_ of 8.1 μM; the inhibitory effect of AEA on cromakalim-induced K_ATP_ currents is independent of CB receptors and of G_i/o_-coupled receptors, as manifested by the ineffectiveness of CB receptor antagonists and pertussis toxin, respectively, to prevent AEA-induced block. Furthermore, inhibitors for AEA’s degradative enzymes amidohydrolase and cyclooxygenase fail to affect the blockade of cromakalim-induced K_ATP_ currents caused by AEA, indicating that the effect of AEA is not mediated by its metabolic products [29]. Evidence provide by Oz et al. [29] thus suggests that AEA may modulate the hormonal maturation process in *Xenopus* oocytes by modulating K_ATP_ channel activity.

AEA is involved in the regulation of cardiovascular function [81]. In addition to Kv channels in ventricular myocytes and vascular smooth muscle cells (see above), myocardial K_ATP_ channels are also functionally modulated by AEA. Contrary to the observations made in pancreatic β–cells [54] and follicle-enclosed oocytes [29] where endocannabinoids suppress native K_ATP_ channel function, Li et al. [44] reported that in isolated rat ventricular myocytes, AEA increases whole-cell K_ATP_ currents induced by dinitrophenol, a mitochondrial uncoupler, in a concentration-dependent manner. Furthermore, the stimulatory effect of AEA on ventricular K_ATP_ currents is reduced by pretreatment of cells with the CB2 receptor antagonist AM630, whereas the CB1 receptor antagonist AM251 has no effect. These data thus suggest that AEA augments ventricular myocardial *I*_KATP_ through a CB2 receptor-dependent pathway [44], which may underlie the antiarrhythmic and cardioprotective action of AEA; by contrast, AEA exerts no effect on another inward rectifier current *I*_K1_ (i.e., cardiac classical inwardly rectifying K^+^ current [82]) in ventricular myocytes. Nevertheless, whether AEA causes an inhibitory effect on myocardial K_ATP_ channels in excised membrane patches remains to be determined. Endocannabinoid production can be induced when the cardiovascular system is functioning under deleterious conditions such as circulatory shock or hypertension; endocannabinoids are also involved in preconditioning by nitric oxide [for a review, see [41]]. Activation by endogenously released AEA under pathophysiological conditions may contribute to the cardioprotection afforded by sarcolemmal K_ATP_ channels. The difference in K_ATP_ channel responses between different cell types to endocannabinoids may be partly attributed to the distinct, tissue-specific molecular (subunit) compositions of K_ATP_ channels or the cellular background in which the channels are expressed.

#### Time course of direct modulation of channel function by endocannabinoids

Across the studies discussed above, the time course for endocannabinoid lipids and analogues to induce the potassium channel-modulating effects is generally slow, with a maximal response achieved after several minutes (usually within 10 min) of continuous drug exposure [e.g., 37,42,44,48,50,51,59,60,66]. Moreover, there is no significant washout of the endocannabinoid effect upon perfusion with drug-free solution, unless the wash solution contains lipid-free BSA (a lipid scavenger) [e.g., 48,51,66]. In almost all of these studies, the experimentation was performed at room temperature, except the study by Gantz and Bean [47], where the experimentation was conducted at 37°C instead. Cannabinoids are lipid-soluble compounds, and dimethyl sulfoxide (DMSO) or 100% ethanol was chosen as a solvent to prepare aliquots of endocannabinoids at millimolar concentrations in these studies, with the final concentration of solvent during experiments consistently ≤ 0.1–0.15%. Gantz and Bean [47] showed that the maximal inhibitory effect of 2-AG on the fast inactivating A-type K^+^ current *I*_A_ could be measured within 1 min of drug exposure (in contrast to ~5-12 min typically seen in other studies). The more prompt response to endocannabinoids observed by Gantz and Bean [47] may be attributed, in part, to the higher temperature (37°C instead of room temperature) at which their study was carried out.

### Endocannabinoid interaction with other ion channels

AEA can modulate the functions of ion channels other than potassium channels, such as TRP vanilloid type 1 (TRPV1) channels [83], 5-HT_3_ receptors (5-HT_3_R) [84], nicotinic acetylcholine receptors (nAChRs) [85], glycine receptors (GlyRs) [86], and Ca_V_ [87,88] and voltage-gated Na^+^ (Na_V_) [89,90] channels, in a manner independent of known cannabinoid receptors [2,4,19,30,91]. Several studies are reviewed below to exemplify that, besides potassium channels, multiple ion channel types belonging to other ion channel families can also serve as molecular targets of endocannabinoids, which collectively manifests the relevance of direct modulation of various ion channels in mediating the biological functions of endocannabinoids.

#### TRPV1 channels

The TRP channel superfamily of nonselective, ligand-gated cation channels is involved in numerous physiological functions such as thermo- and osmosensation, smell, taste, vision, hearing pressure or pain perception [for a review, see [92]]. The endocannabinoid AEA is structurally related to capsaicin (the pungent compound from chili peppers), the agonist for TRPV1 channels. It has been demonstrated by Zygmunt et al. [83] that AEA induces vasodilation in isolated arteries in a capsaicin-sensitive manner and that the AEA effect is accompanied by release of calcitonin-gene-related peptide (CGRP), a vasodilator peptide. This vasodilatory action of AEA is abolished by a CGRP receptor antagonist but not by the CB1 receptor antagonist SR141716A; moreover, CB1 and CB2 receptor agonists do not reproduce vasodilation caused by AEA [83]. Additionally, AEA concentration-dependently elicits capsazepine (a TRPV1 channel antagonist)-sensitive currents acquired from cells overexpressing cloned TRPV1 channels in both whole-cell and excised patch modes. These findings thus suggest that AEA induces peripheral vasodilation by activating TRPV1 channels on perivascular sensory nerves independently of CB1 receptors and consequently causing the release of CGRP [83]. AEA and other structurally related lipids may act as endogenous TRPV channel agonists or modulators to regulate various functions of primary sensory neurons such as nociception, vasodilation, and neurogenic inflammation.

#### Voltage-gated calcium and sodium channels

Low voltage-activated or T-type calcium channels, encoded by the Ca_V_3 gene family, regulate the excitability of many cells, including neurons involved in nociceptive processing, sleep regulation and the pathogenesis of epilepsy; they also contribute to pacemaker activities [for a review, see [93]]. The whole-cell currents of both cloned (α_1G_, α_1H_ and α_1I_ subunits) and native T-type calcium channels are blocked by submicromolar concentrations of AEA; this effect is prevented by inhibition of AEA membrane transport with AM404, suggesting that AEA acts intracellularly [87]. AEA concentration-dependently accelerates inactivation kinetics of T-type calcium currents, which accounts for the reduction in channel activity [87]. The inhibitory action of AEA on these Ca_V_ channels is independent of CB1/CB2 receptors and G proteins, and the inhibition is preserved in the excised inside-out patch configuration, implying a direct effect; furthermore, AEA has little effect on membrane capacitance, reflecting that its effects are unlikely attributed to simple membrane-disrupting mechanisms [87]. Accordingly, it is postulated that AEA may directly target and modulate T-type calcium channels to elicit some of its pharmacological and physiological effects.

High voltage-activated, dihydropyridine-sensitive L-type calcium channels are involved in excitation-contraction coupling in skeletal, smooth, and cardiac myocytes as well as the release of neurotransmitters and hormones from neurons and endocrine cells [93,94]. It has been demonstrated via biochemical assays that AEA is able to displace specific binding of L-type calcium channel antagonists to rabbit skeletal muscle membranes in a concentration-dependent manner, with the IC_50_ around 4–30 μM [88], supporting a direct interaction between AEA and L-type calcium channels. Furthermore, AEA (1–10 μM) suppresses the whole-cell currents of both native Na_V_ and L-type calcium channels in rat ventricular myocytes in a voltage‐ and pertussis toxin‐independent manner [89], indicating that the inhibitory effect of AEA does not require activation of G_i/o_ protein-coupled receptors like CB1 and CB2 receptors. Direct inhibition of Na_V_ and L-type Ca_V_ channel function may account for some of the negative inotropic and antiarrhythmic effects of AEA in ventricular myocytes [89].

#### Glycine receptors

GlyRs belong to the Cys-loop, ligand-gated ion channel superfamily that comprises both cationic receptors such as nAChRs and 5-HT_3_Rs and anionic receptors such as γ-aminobutyric acid (GABA) type A receptors (GABA_A_Rs) and GlyRs [95].

GlyRs are distributed in brain regions involved in pain transmission and reward, and they are thought to play a role in the analgesia process and drug addiction [96]. Recent studies have shown that GlyRs are an important target for cannabinoids in the central nervous system. AEA, at pharmacologically relevant concentrations (<1 μM), directly potentiates the function of recombinant GlyRs expressed in oocytes and native GlyRs present in acutely isolated rat ventral tegmental area (VTA) neurons through an allosteric, CB1 receptor-independent mechanism [86]. The stimulatory effect of AEA on GlyRs is selective, as neither the GABA-activated current in VTA neurons nor the recombinant α2β3γ2 GABA_A_R current in oocytes is affected by AEA treatment [86].

#### Nicotinic acetylcholine receptors

The homomeric α7 receptor is one of the most abundant nAChRs in the nervous system and it is involved in pain transmission, neurodegenerative diseases, and drug abuse [for a review, see [97]]. The endocannabinoid AEA has been shown to inhibit nicotine-induced currents in *Xenopus* oocytes expressing cloned α7 nAChRs; the inhibition is concentration-dependent with an IC_50_ of 229.7 nM and noncompetitive [85]. In addition, pharmacological approaches using specific inhibitors uncovered that the inhibitory effect of AEA on α7 nAChRs does not require CB receptor activation, G protein signaling, AEA metabolism, or AEA membrane transport, suggesting that AEA inhibits the function of neuronal α7 nAChRs expressed in *Xenopus* oocytes via direct interactions with the channel [85]. AEA is structurally similar to other fatty acids such as arachidonic acid and prostaglandins; it is possible that AEA and other fatty acids that are capable of modulating nAChRs [98] share some common mechanisms of action to control the channel function.

### Possible mechanisms involved in canonical cannabinoid receptor-independent modulation of ion channels by endocannabinoids

It is well established that potassium channels are important players in controlling the duration, frequency, and shape of action potentials, thereby controlling cell excitability [22]. As described above, the endocannabinoid AEA is capable of exerting CB1/CB2 receptor-independent functional modulation of a variety of potassium channels, including native BK [37], *I*_to_ [44], delayed rectifier [50,51], K_ATP_ [29] and TASK-1 [66] channels, as well as cloned TASK-1 [66], Kv4.3 [42], Kv3.1 (delayed rectifier being converted into A-type current) [61], Kv1.2 [48], and Kv1.5 [59,60] channels (see Table 1). Moreover, native neuronal *I*_A_ [47], pancreatic β–cell delayed rectifier and K_ATP_ [54], and atrial myocardial delayed rectifier [59] potassium channels are subject to modulation by another endocannabinoid 2-AG, also in a CB receptor-independent manner (see Table 1). Likewise, for TRPV1 channels [83], ligand-gated ion channels such as cloned and native GlyRs [86], cloned α7 nAChRs [85] and native 5-HT_3_Rs [84], plus voltage-gated ion channels such as native Na_V_ [89], native L-type Ca_V_ [88,89], and native and cloned T-type Ca_V_ [87] channels, the functional modulation elicited by AEA does not require activation of CB receptors (see Table 2).

Interestingly, in the majority of studies reviewed in this article, the CB receptor-independent modulatory effects of AEA are induced only when endocannabinoids are introduced extracellularly to the ion channel targets that include cloned Kv1.2, hKv1.5, Kv3.1, and hKv4.3 channels heterologously expressed, and native delayed rectifier Kv channels in aortic vascular smooth muscle cells and cortical astrocytes [42,48,50,51,59,61], whereas in several reports endocannabinoids only alter ion channel function when administrated at the cytoplasmic side of the membrane [54,60,87]. These observations imply the presence of distinct interaction sites or mechanisms of action, which may be attributable to differences in the types of ion channels or endocannabinoids investigated, cell models/cellular environments channels being exposed to, or experimental protocols adopted.

On the other hand, although membrane environment seems to be critical for the regulation of signal transduction pathways triggered by G protein-coupled receptors like CB1 receptors [for a review, see [28]], current evidence does not support an involvement of changing membrane fluidity or altering lipid bilayer properties [99] in mediating the CB receptor-independent actions of AEA on ion channels [42,51,66]. Besides, it is worth noting that, unlike 2-AG, which is entirely localized in lipid rafts in dorsal root ganglion cells, most of AEA (~70%) is found in non-lipid raft fractions of the membrane [100]. It is therefore less likely that changes in membrane fluidity serve as a primary mechanism of action responsible for AEA’s CB receptor-independent effects.

Lipid signals like endocannabinoids and structurally related fatty acids may modify gating of voltage-gated ion channels through a direct action on the channel (or on some regulatory component closely associated with the channel) via a membrane lipid interaction [42,47,101,102]. A model for direct interactions between ion channel proteins and endocannabinoids is further supported by identification of specific residues in several channel proteins crucial for the CB receptor-independent modulatory actions exerted by endocannabinoids [59,60,103]. For example, AEA may directly interact with, and in turn be stabilized by, a ring of hydrophobic residues formed by valine 505 and isoleucine 508 in the S6 domain around the ion conduction path of the hKv1.5 channel, thereby plugging the intracellular channel vestibule as a high potency open-channel blocker and suppressing the channel function [60]. Molecular dynamic simulations have also helped reveal novel interactions between AEA and the TRPV1 channel on a molecular level, suggesting that AEA enters and interacts with TRPV1 in a location between the S1-S4 domains of the channel via the lipid bilayer [91].

### Conclusions and perspectives

The endocannabinoid system serves as an important homeostatic regulator in essentially all organ systems and additionally is involved in pathophysiological conditions where its activation renders symptom relief or protection against the progression of certain disorders. Endocannabinoids and other membrane-derived lipids are important signaling molecules; apart from acting through specific membrane receptors, lipid mediators are known to directly target ion channels to deliver their functional effects [101]. Indeed, the endocannabinoid AEA is not selective for the canonical CB receptors, and its modulatory actions appear to be more complex than originally thought [for a review, see [19]]. Existing evidence suggests that various types of potassium channels, voltage-gated Na^+^ and Ca^2+^ channels, TRP channels, and ligand-gated ion channels are directly modulated by AEA (see Tables 1 and 2). The CB receptor-independent effects elicited by AEA on ion channels include both functional inhibition and stimulation, presumably defined by the channel types and the underlying mechanisms of action. AEA may interact with its channel targets at their intracellular [54,87] or extracellular side [42,48,50,51,59,61], or plug the channel pore from the cytoplasmic side as an open-channel blocker [60]. AEA has been implicated in diverse physiological and pathophysiological processes, and both AEA and another endocannabinoid 2-AG have anxiolytic, analgesic and neuroprotective effects [28,104]. Considering the importance of the endocannabinoid system in human physiology and the therapeutic implications of cannabinoids and ion channels in a vast number of medical conditions, further investigation into the molecular mechanisms underlying the interactions between different types of cannabinoids and their ion channel targets is needed for future development of novel cannabinoid-based therapeutic strategies with improved specificity.
